# Survival with chronic myeloid leukaemia after failing milestones

**DOI:** 10.1038/s41375-023-02028-2

**Published:** 2023-09-19

**Authors:** Michael Lauseker, Rüdiger Hehlmann, Andreas Hochhaus, Susanne Saußele

**Affiliations:** 1 Institut für Medizinische Informationsverarbeitung, Biometrie und Epidemiologie – IBE, Medizinische Fakultät, LMU München, München, Germany; 2 ELN Foundation, Weinheim, Germany; 3 Medizinische Fakultät Mannheim, Universität Heidelberg, Mannheim, Germany; 4 Klinik für Innere Medizin II, Universitätsklinikum Jena, Comprehensive Cancer Center Central Germany, Jena, Germany

**Keywords:** Chronic myeloid leukaemia, Molecularly targeted therapy

## Abstract

Therapy after failing response milestones in CML is controversial. Risks associated with comorbidities, drug toxicities or transplantation may preclude switching to another tyrosine kinase inhibitor (TKI) or other treatments. No information on long-term survival of failing patients is available. To systematically analyse survival after reaching, or not reaching, response milestones, 1342 patients from CML-study IV with newly diagnosed CML in chronic phase and regular molecular tests were studied. Landmark survival analyses were done by <0.1%, 0.1–1%, >1–10% and >10% BCR::ABL1^IS^ at 3, 6, 12 and 24 months up to 14 years. 10- to 12-year survival of patients who failed the failure milestones (>10% BCR::ABL1^IS^ at 6 months, >1% BCR::ABL1^IS^ at 12 months) ranged around 80%, 10% less than in responding patients. These results suggest revision of milestones. Age (more or less than 60 years) had no major impact on survival differences, but on hazard ratios and CML-specific survival. Switching to alternative therapies, which was observed in 26.9% of the patients, did not change the main results. The data show that TKI-treated patients not reaching failure milestones still may derive benefit from continuing TKI-treatment and provide a basis for individualised decisions, if failing patients are confronted with risks of alternative treatments.

## Introduction

Controversies in CML management have surfaced regarding treatment after failing response milestones. Response milestones, since their definition and first publication by the European LeukemiaNet (ELN) in 2006 [1–3], have been a cornerstone for therapeutic decisions in CML-patients treated with tyrosine kinase inhibitors (TKI). Patients who reach less than 10% BCR::ABL1 by 3 months have high probabilities of a normal life expectancy [4, 5]. Also patients reaching response milestones at later times may still have excellent survival perspectives. Patients failing milestones (>10% BCR::ABL1 by 6 months, >1% BCR::ABL1 by 12 months or later) have a worse prognosis and are generally switched to an alternative TKI. In some patients switching may be problematic due to risks associated with drug toxicities, comorbidities or transplantation [6, 7]. In these patients, long-term observations have suggested that survival of patients failing milestones at 6 or 12 months and not switching TKI treatment was indeed worse than in patients who reached the milestones, but less than expected [8]. In CML-study IV, the difference of survival (OS) at 10 years between patients reaching, or not reaching, the warning milestone 1% BCR::ABL1 at 6 months was 6.4% across all treatment arms [9]. This implies that survival of patients not reaching, or failing, milestones may still be at a level permitting continuation of TKI-treatment, if there are concerns regarding risks of alternative treatments. It is the purpose of this analysis to follow up on these observations by systematically comparing TKI-treated CML patients in chronic phase (CP) who met, or did not meet, the various response milestones and quantifying their long-term survival.

## Patients and methods

CML-study IV [9] is a randomised 5-arm study comparing newly diagnosed patients with CML in CP treated with imatinib 400 mg/day versus imatinib in combination with low-dose cytarabine or with interferon alpha simultaneously or consecutively versus imatinib 800 mg/day (*n* = 1536). Recruitment was from July 2002 through March 2012. Patients were followed for up to 14 years. No experimental arm turned out to be superior to standard imatinib at 400 mg/day, but the study showed high rates of stable deep molecular responses across study arms [10, 11]. For this analysis, only patients were selected who received imatinib as initial treatment and had regular molecular tests done (*n* = 1342).

In 372 patients (26.9%), imatinib, as foreseen in the study-protocol in the case of resistance or intolerance, was switched to another therapy, mostly to dasatinib and nilotinib. Seven patients were switched to bosutinib, 5 to ponatinib and 58 to more than one TKI. Seventy-eight patients were transplanted in first CP. Censoring at the time of switching did not change the main results of this analysis.

Definitions and end points: Definitions followed the ELN-recommendations [3]. OS was defined as the time between diagnosis and death resulting from any cause. Death unrelated to CML was defined as death without prior progression and unrelated to CML-therapy. Death due to CML was stratified according to the European treatment and outcome study-long-term-survival score [12]. All living patients were censored at the time of their last visit.

Cytogenetic and molecular analyses: Cytogenetic and molecular diagnostics were performed as described [4, 9, 13]. Testing for residual BCR::ABL1^IS^ transcripts was done using quantitative reverse-transcription polymerase chain reaction with defined conversion factors for equivalence of tests according to International Scale (IS). The nomenclature followed the HGNC-recommendations [14]. Testing was restricted to patients expressing e13a2 and/or e14a2 transcripts. Equivalence of cytogenetic and molecular tests has been shown [15].

Statistical evaluations: Landmark analyses were carried out for four time points (3, 6, 12, and 24 months) to estimate conditional overall survival. Thus, all results have to be interpreted under the condition of still being alive at the respective landmark and therefore ignoring early mortality before the landmark. We performed two sensitivity analyses: Firstly, patients were censored at the time of treatment switch. This was done to assess the stability of the results presented here. Secondly, age was included as a metrical covariate into the models (instead of a dichotomisation at the 60 years cut-off). *P* values less than 0.05 were considered significant. This analysis was not specified in the study protocol; therefore, it must be seen as exploratory. All analyses were done in R 4.2.2 (R foundation). Landmark curves were estimated using the survival package. The differences between the conditional survival probabilities were plotted using the rms package.

## Results

### Response levels ≤0.1%, >0.1–1%, >1–10% and, >10% BCR::ABL1^IS^

To systematically analyse survival after reaching, or not reaching, response milestones, molecular and survival data from CML-study IV were used. Molecular tests were done in a total of 1342 imatinib-treated patients, of these in 805 patients at 3, in 891 at 6, in 861 at 12, and in 755 at 24 months after diagnosis. Of particular interest was the survival of patients who failed the failure milestones 10% BCR::ABL1^IS^ at 6 months and 1% BCR::ABL1^IS^ at 12 months. In clinical practice, the intermediary levels between >0.1% and 1% and between >1% and 10% BCR::ABL1^IS^ may also be of interest.

**Fig. 1:**
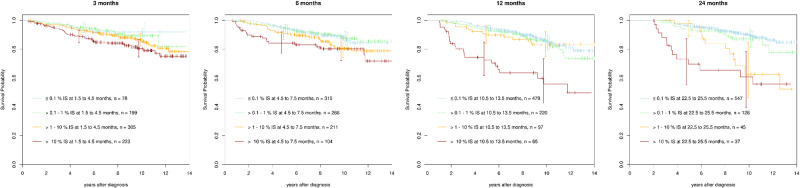
Landmark survival analyses at 3, 6, 12 and 24 months according to reaching, or not reaching, 0.1%, >0.1–1%, >1–10% and >10% BCR::ABL1^IS^. Patients reaching 0.1% and 1% BCR::ABL1^IS^ at 3, 6, 12 and 24 months had a 10-year OS of close to 90%. Patients not reaching 10% BCR::ABL1^IS^ at 3 and 6 months had a 10-year survival of 80% despite significant inferiority compared with patients reaching 0.1% and 1%. Patients not reaching 10% BCR::ABL1^IS^ at 12 months had a marked increase in survival inferiority confirmed at 24 months. 10-year OS dropped to 55%. Likewise, 10-year survival of patients failing >1-10% remained stable around 90% until 12 months, but dropped to 60% at 24 months.

Landmark survival analyses were carried out according to response levels of ≤0.1%, >0.1–1%, >1–10% and >10% BCR::ABL1 at 3, 6, 12 and 24 months (Fig. 1). Conditional survival after failing molecular response milestones was evaluated. Censoring for switching therapy did not change the main results (data not shown).

Whereas survival after reaching, or not reaching, >0.1–1% BCR::ABL1^IS^ was not much different from survival after reaching 0.1% BCR::ABL1^IS^ at all time points, survival after reaching, or not reaching, >1–10% BCR::ABL1 requires a more differentiated look. Landmark analyses up to month 12 show a survival similar to 1% BCR::ABL1^IS^. The landmark analysis at month 24 shows a survival similar to 1% BCR::ABL1^IS^ up to year 6 when survival drops to the level of >10% BCR::ABL1^IS^.

Patients not reaching 10% BCR::ABL^IS^ at 3 and 6 months had a significantly inferior conditional OS compared to those reaching 1% or 0.1% BCR::ABL^IS^, but a 10-year OS of about 80%. At the 12-months landmark, inferiority of conditional OS compared to patients with lower BCR::ABL^IS^ levels increased markedly and 10-year OS dropped to 55%. The data suggest >10% BCR::ABL^IS^ at 12 months as a more meaningful failure milestone compared to the 3- and 6-months landmarks which rather are warning milestones.

**Fig. 2:**
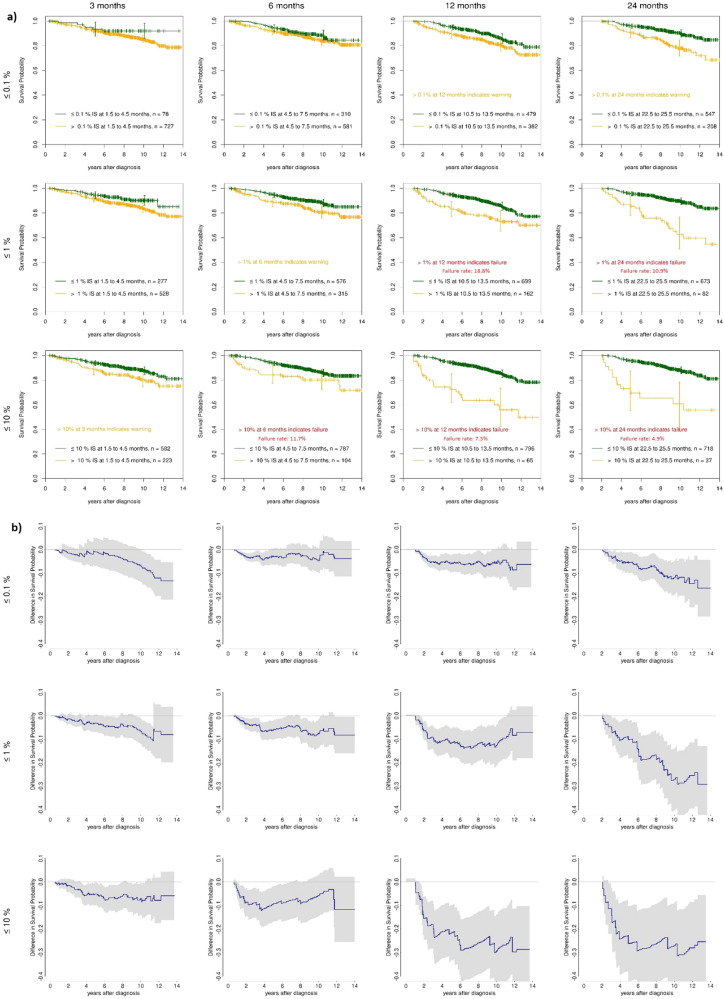
Differences of conditional survival between reaching and not reaching response milestones. **a** Landmark survival analyses (all patients) at 3, 6, 12 and 24 months according to reaching, or not reaching, molecular response levels at 0.1%, 1% and 10% BCR::ABL1^IS^, **b** calculated conditional survival differences according to molecular response levels of 0.1%, 1% and 10% BCR::ABL1^IS^ at 3, 6, 12 and 24 months with confidence intervals.

As the ELN recommendations use dichotomisations, we had a closer look on those (Fig. 2a) and the resulting differences in conditional overall survival (Fig. 2b).

### Response level 0.1% BCR::ABL1^IS^

The survival differences (conditional overall survival, OS) between patients reaching, or not reaching, 0.1% BCR::ABL1 at 3 or 6 months (line 1 of Fig. 2a) were always below 10% in the first 10 years (see line 1 of Fig. 2b). We did not find significant differences regarding the hazard ratios. Conversely, not reaching, 0.1% BCR::ABL1^IS^ at 12 and 24 months resulted in significant differences in hazard ratios of 0.63 (*p* = 0.010) respectively 0.43 (*p* < 0.001). Both indicate a warning. For the 12 months landmark, we observed constant, yet small differences in survival below 10% in the conditional probabilities. For the 24 months landmark, the differences between both groups reached 14.4% (pointwise 95%-confidence interval (CI): 4.5–24.5%) at 12 years.

### Response level 1% BCR::ABL1^IS^

The conditional OS differences between patients reaching, or not reaching, 1% BCR::ABL1^IS^ at 3 and 6 months were comparatively small with maxima of 10.7% and 8.2% both at about 12 years (line 2 of Fig. 2b). Not reaching 1% at 12 or 24 months is considered a failure. Here, the differences occurred earlier. For the 12 months landmark, the difference was already 10% at 3 years. For the 24 months landmark, the differences reached up to 29.0% (95%-CI: 12.8–45.2%). The hazard ratios for all four landmarks were significantly different from one and ranged from 0.26 (24 months, *p* < 0.001) to 0.63 (3 months, *p* = 0.031).

### Response level >10% BCR::ABL1^IS^

The conditional OS differences between patients reaching or not reaching 10% BCR::ABL1^IS^ at 3 months (line 3 of Fig. 2a) were again below 10% at maximum (7.8% at about 12 years). For the other landmarks, where not reaching 10% BCR::ABL1^IS^ is considered a treatment failure, we found larger and earlier differences (Fig. 2b, line 3). These differences grew to about 30% for the 12- and 24-months landmarks, both at around 6 years. Similar to the 1% level, the hazard ratios for all four landmarks were significantly different from one and ranged from 0.22 (24 months, *p* < 0.001) to 0.61 (3 months, *p* = 0.015).

### Slow responders and impact of residual disease

Figure 2a also shows that the proportions of patients failing the failure milestones 1% BCR::ABL1^IS^ by 12 months and 10% BCR::ABL1^IS^ by 6 months decreased during the observation interval from 18.8% at 12 months to 10.9% at 24 months at the 1% response level (line 2), and from 11.7% at 6 months to 7.5% at 12 months and further to 4.9% at 24 months at the 10% level (line 3) assuming that a substantial proportion of initially failing patients responded later (slow responders) [9]. It is apparent that survival is dependent on the amount of residual disease still present at subsequent time points. Not to have reached <1% BCR::ABL1^IS^ at 12 months (line 2), or <10% at 6 and 12 months (line 3) is less impacting on OS than to have not reached this goal at 24 months.

### Impact of age

**Fig. 3:**
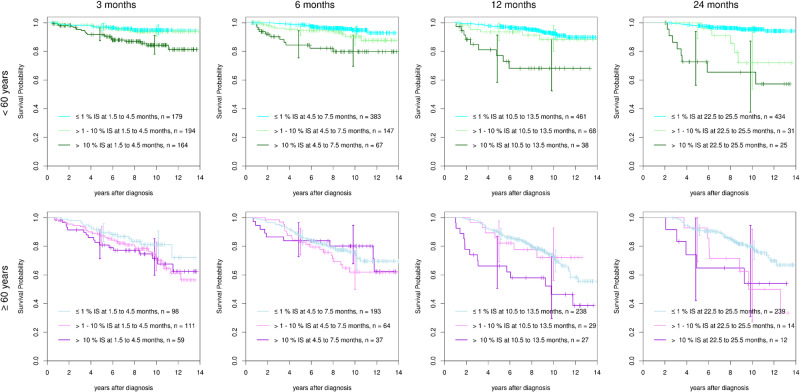
Landmark survival analyses (<60 years, line 1; ≥60 years, line 2) at 3, 6, 12 and 24 months according to reaching, or not reaching 1%, >1–10% and >10% BCR::ABL1^IS^. The age groups show clear survival differences, whereas reaching milestones is similar. Not reaching 10% BCR::ABL1^IS^ at 12 months is associated with a drop of 10-year OS in both age groups.

The respective analyses for all patients stratified by age more or less than 60 years are shown in Fig. 3. Although the analyses show similar results according to reaching or not reaching milestones in both age groups, the underlying survival was rather different between younger and older patients translating to large differences in the hazard ratios for early death. Using Cox models with interaction effects, we found hazard ratios of 4.0 (95% CI: 2.0–8.1) for the younger patients, but only 0.9 (95%-CI: 0.4–1.8) for the elderly at 10% BCR::ABL1^IS^ by 6 months. Similar differences were observed for the other milestones that indicated a failure, and, to a slightly smaller degree, for the warnings. The reasons for early death in the younger patients were mostly CML-related, whereas the elderly primarily died of other reasons as determined earlier [12]. In a sensitivity analysis, age was considered a metrical covariate. As the main conclusions regarding the landmarks were the same as before, we decided to stay with the dichotomisation for the sake of the visualisation.

### Impact of early cytogenetic response on survival

**Fig. 4:**
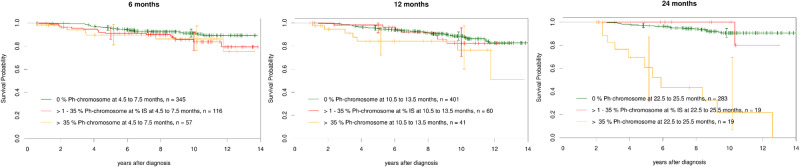
Landmark survival analyses according to achievement of complete, partial, and less than partial cytogenetic remission at 6, 12 and 24 months. All 3 cytogenetic resüonse groups show a 10-year survival of close to 90%. A small group of patients (*n* = 19, 5.9% of 321) did not reach a partial cytogenetic remission at 24 months and had an inferior survival with a 5-year survival of 60% and a 10-year survival of 25%.

When cytogenetic analyses were used to define milestones, similar survival results were seen as with the corresponding molecular tests (Fig. 4). Cytogenetic analyses were available in 518 patients at 6 months, in 502 patients at 12 months and in 321 patients at 24 months. The differences in conditional OS between patients achieving complete, partial, or less than partial cytogenetic remissions were not significant at months 6 and 12. At 24 months patients that did not achieve at least a partial cytogenetic remission showed an inferior conditional OS compared to both other groups.

## Discussion

Our data show that CML-patients derive benefit from TKI treatment even if milestones defining failure are not reached. TKI-treated patients not reaching the failure milestones 1% BCR::ABL1^IS^ by 12 months and 10% BCR::ABL1^IS^ by 6 months still have a 10-to-12-year conditional OS of 80% which is 10% less than that of responders. This data may help decide on treatment continuation when an alternative treatment carries risks due to patients‘ comorbidities, adverse drug effects or transplantation. Treatment can be individualised and adapted to patients‘ needs by taking survival under continued TKI-treatment and risk of the new treatment into consideration. In view of these data definitions of failure and warning milestones may have to be revised.

Our data compare well with the 60% 15-year OS observed with 131 CP-patients who did not reach MMR within 2 years of TKI treatment [16], but patients not reaching at least partial cytogenetic remission within 24 months (19 of 321) may have a poorer survival. To verify a survival difference between these subgroups, a comparison between studies would be needed that takes risk profiles, cytogenetics [17] and other prognostic factors into consideration.

If the 10% BCR::ABL1^IS^ response level is not reached by 12 or 24 months, the survival difference to responders increases to about 30% which still may be an acceptable outcome in the absence of reasonable alternatives.

The impact of intermediate response levels >1–10% BCR:ABL1^IS^ on survival shows the limitations of a dichotomous evaluation approach and points to the need of individualised interpretation.

The strength of the data comes from the number of patients, the study protocol of CML-study IV [9] asking for regular molecular analyses of all patients over the entire study duration, and from the follow up of up to 14 years.

The survival differences between patients reaching and not reaching the failure milestones confirm the value of the response milestones [1, 2], but failure milestones may have to be adapted to the new more mature data.

If the data were analysed according to age more or less than 60 years, differences between the age groups were noted. Younger patients not reaching the failure milestones were more likely to die early, mostly of progression, whereas reaching the milestones is less important for the elderly who died predominantly of other reasons [12].

A limitation of the data is that imatinib was the primary TKI in CML-study IV. As differences between patients with and without failures or warnings were similar when patients were censored at the time of switching, we can conclude that the milestones were reasonable in both scenarios. The design of CML study IV precludes a comparison of imatinib to 2nd generation TKI after treatment failure.

The molecular data are confirmed by cytogenetic data. Cytogenetics were increasingly replaced by molecular tests during the study, but as evident from Fig. 4, there still were sufficient patients with cytogenetic analyses available to show that results with cytogenetically and molecularly defined milestones were similar.

In conclusion, TKI-treated patients not reaching failure milestones still may derive benefit from continuing TKI-treatment with acceptable survival probabilities showing that TKI have a beneficial effect even if milestones are not reached. The definition of milestones may have to be revised. Individualisation of therapy according to comorbidity, effectivity and treatment goals is desirable.

## Data Availability

Authors are committed to sharing access to patient-level data and supporting clinical documents with qualified external researchers upon request. These requests are reviewed and approved by an independent review panel based on scientific merit. All data provided are anonymized to respect the privacy of patients who have participated in the trial consistent with applicable laws and regulations.
